# Enhancer variants: evaluating functions in common disease

**DOI:** 10.1186/s13073-014-0085-3

**Published:** 2014-10-28

**Authors:** Olivia Corradin, Peter C Scacheri

**Affiliations:** Department of Genetics and Genome Sciences, Case Western Reserve University, Cleveland, OH 44122 USA; Case Comprehensive Cancer Center, Case Western Reserve University, Cleveland, OH 44106 USA

## Abstract

Gene enhancer elements are noncoding segments of DNA that play a central role in regulating transcriptional programs that control development, cell identity, and evolutionary processes. Recent studies have shown that noncoding single nucleotide polymorphisms (SNPs) that have been associated with risk for numerous common diseases through genome-wide association studies frequently lie in cell-type-specific enhancer elements. These enhancer variants probably influence transcriptional output, thereby offering a mechanistic basis to explain their association with risk for many common diseases. This review focuses on the identification and interpretation of disease-susceptibility variants that influence enhancer function. We discuss strategies for prioritizing the study of functional enhancer SNPs over those likely to be benign, review experimental and computational approaches to identifying the gene targets of enhancer variants, and highlight efforts to quantify the impact of enhancer variants on target transcript levels and cellular phenotypes. These studies are beginning to provide insights into the mechanistic basis of many common diseases, as well as into how we might translate this knowledge for improved disease diagnosis, prevention and treatments. Finally, we highlight five major challenges often associated with interpreting enhancer variants, and discuss recent technical advances that may help to surmount these challenges.

## Introduction

**Figure 1:**
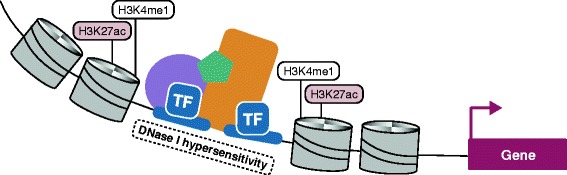
**Model of enhancer function.** Transcriptional enhancer elements are noncoding stretches of DNA that regulate gene expression levels, most often in *cis*. Active enhancer elements are located in open chromatin sensitive to DNase I digestion and flanked by histones marked with H3K4me1 and H3K27ac. Enhancers are often bound by a number of transcription factors (TF), such as p300 (blue). Mediator and cohesin are part of a complex (orange, green and purple) that mediates physical contacts between enhancers and their target promoters.

Transcriptional enhancer elements are noncoding stretches of DNA that have a central role in controlling gene expression programs in cells. Rather than on-off switches, enhancers are hypothesized to function as transcriptional rheostats to fine-tune target transcript levels. Higher-order three-dimensional organization of chromatin facilitates physical interactions between enhancers and their target promoters. Interactions between enhancers and their targets may occur on the same chromosome (in *cis*) or on different chromosomes (in *trans*) (Figure 1) [1-3]. In any given mammalian cell type, the number of putative enhancer elements ranges from 50,000 to 100,000, and therefore far exceeds the number of protein-coding genes.

**Figure 2:**
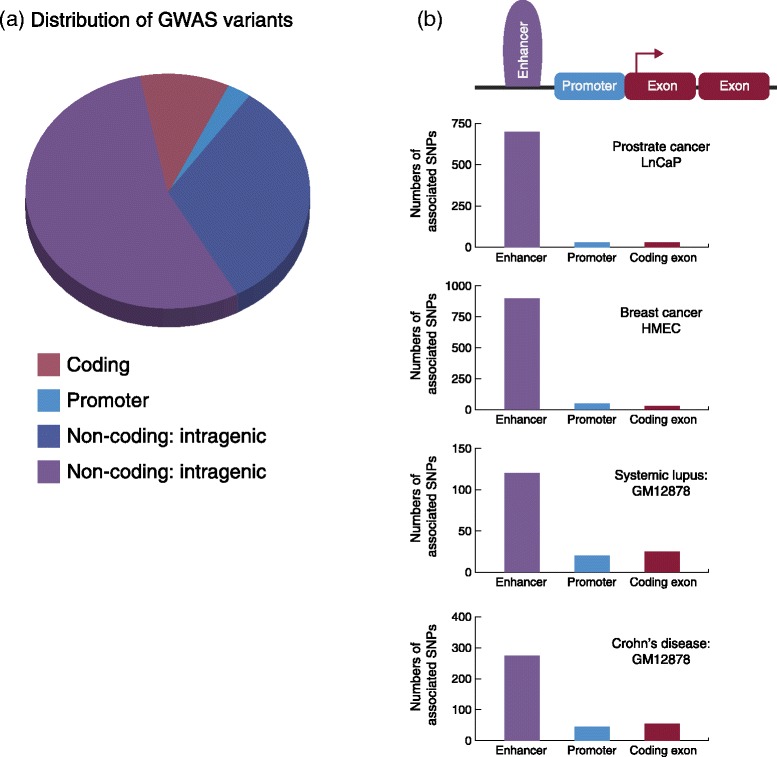
**Enrichment of genome-wide association study variants in putative enhancer elements. (a)** Number of disease-associated variants (identified in the National Human Genome Resource Institute’s genome-wide association study (GWAS) catalog) that lie in protein-coding regions (red), promoters (blue), noncoding intragenic regions (light purple) and noncoding intergenic regions (dark purple). **(b)** Examples of four different common diseases, showing the number of associated single nucleotide polymorphisms (SNPs) that lie in putative enhancers, promoters and exons [6-8]. Putative enhancer elements were defined by chromatin features in each of the four indicated cell types.

In the last decade, more than 1,900 genome-wide association studies (GWASs) have been published, identifying loci associated with susceptibility to over 1,000 unique traits and common diseases [4]. With the eventual goal of finding new therapies and preventative measures for common diseases, efforts are now focused on determining the functional underpinnings of these associations. Several groups have associated GWAS risk variants, mostly SNPs, with newly annotated cell-type-specific gene enhancer elements identified through epigenomic profiling studies. These enhancer variants probably play an important part in common disease susceptibility by influencing transcriptional output. Of all the genetic risk variants discovered to date, the number that impact enhancer function is estimated to far exceed the number that affect protein-coding genes or disrupt promoter function (Figure 2). Additionally, disease-associated variants in noncoding regions, particularly those that lie in cell-type-specific enhancer elements, have been estimated to explain a greater proportion of the heritability for some disorders than variants in coding regions [5]. This review focuses on the identification and interpretation of disease-associated variants that affect enhancer function. We consider the latest approaches for evaluating enhancer variants and identifying their gene targets, and highlight successful cases in which risk variants have been shown to alter gene expression by disrupting enhancer function. In addition, we discuss the remaining challenges to delineating the impact of noncoding variants, such as the identification of enhancer activity, validation of causal variants and identification of responsible genes. Future efforts to surmount these challenges should help to remove the barrier between the discovery of disease associations and the translation of this knowledge for improved diagnosis and treatment of many common diseases.

## Genetic risk variants are enriched in cell-type-specific enhancer elements defined by signature chromatin features

The locations of enhancer elements coincide with DNase I hypersensitive regions of open chromatin flanked by nucleosomes marked with the mono- and/or di-methylated forms of lysine 4 at histone H3 (H3K4me1/2) [9,10]. Enhancers can be active or repressed, and each state generally correlates with the presence of additional histone marks, such as H3K27ac and H4K16ac which are associated with active chromatin, or H3K27me3 and H3K9me3 which are associated with repressed chromatin [11-14]. Active enhancers are bi-directionally transcribed and capped at their 5′ end [15,16]. Most enhancer elements are located in introns and intergenic regions, although some are exonic [17-19]. Relative to promoters, the distribution of enhancers across the epigenome is highly cell-type specific. Some of the first studies to associate GWAS variants with enhancer elements integrated genetic risk variants with regulatory element maps generated through epigenomic profiling (using chromatin immunoprecipitation combined with massively parallel DNA sequencing (ChIP-seq) and the profiling of DNase I hypersensitive sites (DHSs)) [20-22]. Two major themes emerged from these studies. First, loci with signature enhancer features (DHSs, H3K4me1, H3K27ac) are highly enriched for genetic risk variants relative to other chromatin-defined elements such as promoters and insulators [21]. Second, risk variants preferentially map to enhancers specific to disease-relevant cell types in both cancer and other common diseases [21]. For example, type 2 diabetes-associated variants preferentially map to pancreatic islet enhancers [22-25], and SNPs predisposing to colon cancer are enriched in enhancer elements in colon cancer cells and colon crypts, from which colon cancer is derived [26]. Further assessment of the effects of enhancer risk variants has shown that they can alter transcription-factor-binding sites (TFBSs) and impact the affinity of transcription factors for chromatin, and/or induce allele-specific effects on target gene expression [6,27-40]. These studies illustrate the utility of epigenomic profiling for identifying risk variants that lie in putative enhancer elements and for identifying disease-relevant cell types in which the enhancer variants could exert their regulatory effects.

## Super-enhancers, stretch enhancers, and enhancer clusters: hotspots for genetic risk variants

Four studies recently demonstrated correlations between genetic risk variants and large clusters of active enhancers, similar to locus control regions. These features have been called ‘super-enhancers’ [41,42], ‘stretch enhancers’ [24], ‘multiple enhancers’ [7] and ‘enhancer clusters’ [23], and are similar but not identical between studies, although many of these features overlap. The methods used to identify these clusters are distinct. Super-enhancers, for example, are defined by identifying the top-ranking enhancers on the basis of the levels of associated transcription factors or chromatin marks identified through ChIP studies. Stretch enhancers are defined by stretches of open chromatin more densely and more broadly marked with enhancer-histone modifications than typical enhancers. Despite these differences, many of the defined features overlap. These enhancer clusters are highly cell-type specific and have been proposed to play a predominant role in regulating the cell-type-specific processes that define the biology of a given cell type. Moreover, they are disproportionally enriched for genetic risk variants compared to typical enhancers, and the enrichment is biased toward disease-relevant cell types. These results further support the notion that variants that influence cell-type-specific gene regulation are major contributors to common disease risk, and extend this concept to demonstrate that altering the expression of genes under exquisite regulation can frequently lead to increased risk. Enhancer cluster identification provides a means of detecting highly regulated genes and may help to prioritize noncoding variants that are likely to be functional.

A typical locus identified through a GWAS contains dozens to thousands of SNPs in linkage disequilibrium (LD) with the ‘lead’ SNP that is reported to be associated with the disease in question. Any SNP in LD with the lead SNP may be causal, and the prevailing assumption is that only one is causal. Indeed, this scenario has been reported to be the case for some risk loci involving enhancers [34,43], and there are several examples of Mendelian disorders in which a single enhancer variant causes congenital disease [44-50]. However, it is equally plausible that more than one SNP is causal, particularly at GWAS loci harboring enhancer clusters. In these instances, several variants distributed among multiple enhancers throughout the locus, rather than a single SNP, may combine to affect expression of their gene targets and confer susceptibility to common traits. This has been called the ‘multiple enhancer variant’ (MEV) hypothesis. Corradin and colleagues provided support for the MEV hypothesis for six common autoimmune disorders, including rheumatoid arthritis, Crohn’s disease, celiac disease, multiple sclerosis, systemic lupus erythematosus and ulcerative colitis. The extent of MEVs across additional common diseases is not yet known [7,28,37].

## Interpreting enhancer variants

Given that risk variants lie in cell-type-specific enhancer elements, it is critical to utilize a disease-relevant cell type to identify potential enhancer variants. SNPs associated with a particular disease can be compared to enhancer elements to identify cell types whose active enhancers are disproportionately enriched for disease variants. Variant set enrichment is a permutation-based method that compares the enrichment of genetic risk-variant sets within any functional element (such as H3K4me1-marked putative enhancers) to randomly generated matched genetic risk-variant sets [26,38]. This type of analysis provides an unbiased way of evaluating the utility of a cell type for studying the impact of variants on enhancer elements.

Several computational programs are currently available to integrate chromatin landscapes with GWAS risk variants to identify candidate regulatory SNPs and evaluate their disease-causing potential. These include IGR [38], RegulomeDB [51], HaploReg [52], FunciSNP [53] and FunSeq [54]. These programs are particularly useful for prioritizing SNPs for functional analyses, which may include transcription factor ChIP or electrophoretic mobility shift assays to test whether a given SNP influences a transcription factor’s ability to bind to the enhancer, and *in vitro* and *in vivo* gene reporter assays to test the SNP’s effect on enhancer activity. Additionally, allele-specific expression can be utilized to quantify the impact of enhancer variants within a specific cell type. Finally, DNA editing strategies involving CRISPR/Cas9-based methods can be employed to evaluate the effect of a variant. Following the identification of a functional enhancer variant, the next major challenge is to identify its target and to test the effect of the SNP(s) on target transcript levels. Many enhancer elements are located within 100 kilobases (kb) of the genes that they regulate, but can also be located more than a megabase away, or even on separate chromosomes. Enhancers can regulate genes or long noncoding RNAs. Most genes are regulated by more than one enhancer, and many enhancers regulate more than one target gene [55,56]. The problem is further complicated by our limited knowledge of barrier elements, which block enhancer-gene interactions. The most common method of assigning an enhancer to its nearest gene is inaccurate, with false discovery rate (FDR) estimates ranging from 40% to 73% [55,57]. Refining methods for identifying the nearest gene to looking for the ‘nearest expressed gene’ still results in a high FDR, with 53% to 77% [55,58] of distal elements bypassing the nearest active transcription start site to interact with a distant gene. Clearly, proximity alone cannot be used to accurately identify the target of an enhancer SNP.

## Methods of identifying gene targets of enhancer variants

To identity enhancer targets, DNA fluorescence *in situ* hybridization (FISH) [59,60], as well as chromatin association methods (chromosome conformation capture (3C)) [61], can be employed. These are powerful approaches for evaluating whether a region of interest interacts with a specific genomic target, but they suffer from the limitation that the regions of interest must be pre-specified, that is, they are ‘one-by-one’ approaches. 4C (circular chromosome conformation capture), an extension of 3C, can capture all regions that physically contact a site of interest, without prior knowledge of the regions that contact that site being necessary [62] (that is, a ‘one-to-all’ approach). Higher-throughput methods include carbon-copy chromosome conformation capture (5C, many-to-many), a high-throughput expansion of 3C, Hi-C (all-to-all) and chromatin interaction analysis by paired-end tag sequencing (ChIA-PET) (for detailed comparison of these methods, see reviews [63,64]). These global approaches can enable the identification of loci that directly and indirectly contact enhancers of interest, and can reveal complex interactions in which dozens to hundreds of loci aggregate, so-called transcriptional hubs or enhanceosomes [65]. These types of high-order interactions have been recently described by several studies [55,56,58]. The extent by which they overlap risk loci remains unexplored. Unfortunately, these approaches tend to be expensive and difficult for most labs to execute, and their resolution often prohibits their use for interrogating GWAS loci. Until recently, for example, the resolution of Hi-C was limited to capturing interactions separated by more than one megabase; 5 to 10 times greater than the distance by which most enhancer-gene interactions occur. Despite the limitations, ‘C’-based methods have been implemented to successfully identify targets of enhancer-risk variants and to quantify their functional effects. For example, Cowper-Sal lari and colleagues utilized 3C and allele-specific expression to demonstrate the impact of the breast cancer risk SNP rs4784227 on expression of *TOX3*, thought to have a role in chromatin regulation [38]. Bauer and co-workers utilized 3C to identify *BCL11A* as the gene target of an erythroid enhancer, and then further demonstrated the impact of enhancer variants on transcription factor binding and expression. Gene editing strategies have also been employed to demonstrate that this enhancer is essential for erythroid gene expression [28]. Finally, we highlight a study by Smemo and colleagues in which 4C-seq was used to identify *IRX3* as the target of an enhancer SNP located in intron 1 of the *FTO* gene, which was originally thought to be the target and therefore the causal gene for increased risk of obesity. Functional studies in mice were used to verify that *IRX3* is the most likely causal gene, not *FTO* [30].

## Computational approaches to identify gene targets of enhancer elements

As alternatives to experimental approaches, several groups have developed computational techniques for determining the targets of enhancers [7,16,21,66-70]. These methods are similar in that they compare patterns of regulatory activity across multiple cell types to predict interactions between enhancers and genes. However, they vary significantly in the type of data required to generate enhancer-gene predictions, the specific approaches used to generate and validate the predictions, and their availability (Table 1). The method described by Ernst and colleagues identifies H3K4me1/2 and H3K27ac sites that co-vary with expressed genes within 125 kb of the gene locus, and uses this to predict enhancer-gene interactions [21]. Thurman and co-workers utilized DHS exclusively to predict interactions. Enhancers were assigned to genes by correlating the cross-cell-type DNase I signal at each DHS site with all promoters located within 500 kb [66]. The method developed by Sheffield and colleagues also uses DHS profiles, but additionally incorporates genome-wide expression data [70]. Rather than employing a fixed distance-based model, Shen and colleagues apply chromatin conformation data from Hi-C experiments to guide the association of enhancers to genes marked by H3K4me1, H3K27ac and RNA Pol II [67]. As an alternative to methods based on chromatin structure, Andersson and colleagues leverage cap analysis of gene expression (CAGE) data to correlate transcription at enhancers with gene expression [16]. There are two computational approaches that are publicly available and executable through website or command-line programs: predicting specific tissue interactions of genes and enhancers (PreSTIGE) [7] and integrated methods for predicting enhancer targets (IM-PET) [69]. PreSTIGE identifies enhancers and genes that demonstrate quantitative cell-type specificity based on H3K4me1 and RNA sequencing (RNA-seq), and can process data from human and mouse cell types [68]. IM-PET, like previously discussed methods, considers the proximity of an enhancer to potential gene targets and the correlation of enhancer and promoter activity, along with measures of transcription factor activity and evolutionary conservation.

**Table 1:** **Computational approaches to predicting gene targets of enhancer elements**

**Reference or method**	**Input data required**	**Gene expression**	**Linear model**	**Number of genes with predictions (per cell line)**	**FDR**	**Species**	**Publically available**
Nearest gene	None	Not considered	Nearest gene	NA	~40% to 73%	Any	NA
Nearest expressed gene	Gene expression	Considered	Nearest expressed gene	NA	~53% to 77%	Any	NA
Ernst *et al.* [21]	H3K4me1, H3K4me2, H3K27ac, RNA-seq	Considered	Distance based (125 kb)	NA	Not determined	Human	No
Thurman *et al.* [66]	DNase I hypersensitivity	Not considered	Distance based (500 kb)	NA	Not determined	Human	No
Sheffield *et al.* [70]	DNase I hypersensitivity and RNA-seq	Considered	100 kb	NA	Not determined	Human	Predicted interactions: [http://dnase.genome.duke.edu/]
Shen *et al.* [67]	H3K4me1, H3K27ac, RNA Pol II	Not considered	Topological domain based	5,000 to 8,000	Not determined	Mouse	No
Andersson *et al.* [16]	CAGE	Considered	500 kb	NA	Not determined	Human	No
PreSTIGE [7]	H3K4me1	Considered	Distance (100 kb) and CTCF based	3,000 to 5,000	~13% to 23%	Human	Predicted interactions: http://genetics.case.edu/prestige Method application: http://prestige.case.edu
PreSTIGEouse [68]	H3K4me1	Considered	Distance based (100 kb)	3,000 to 5,000	Not determined	Mouse	Predicted interactions: http://genetics.case.edu/prestige Method application: http://prestige.case.edu
IM-PET [69]	H3K4me1, H3K27ac, H3K4me3 and RNA-seq*	Considered	Distance (2 Mb)	7,000 to 10,000	~1%	Human	Method application: http://www.healthcare.uiowa.edu/labs/tan/IM-PET.html

*Input data utilized in publication, other input options exist. CAGE, cap analysis of gene expression; CTCF, CCCTC-binding factor (zinc finger protein demonstrated to function as an insulator protein); FDR, false discovery rate; Mb, megabases; NA, not applicable; RNA-seq, RNA sequencing.

When the appropriate datasets are available, computational approaches can offer a relatively fast and cost-effective way of identifying putative enhancer-gene interactions in a given cell type. However, they are generally limited to detecting a subset of enhancer-promoter interactions within a given cell type, and none are capable of identifying *trans* interactions. Methods that rely on cell-type specificity or concordant changes in enhancers and genes across cell types may lack the sensitivity to predict interactions for ubiquitously expressed genes or to delineate interactions in domains with a high density of cell-type-specific genes. There is no standard or ‘reference’ dataset to validate the accuracy of gene-enhancer predictions. Thus, each study utilizes a different approach to evaluate accuracy, which makes it difficult to determine which method is most accurate. This necessitates experimental validation of enhancer-gene interactions determined using prediction-based methods. Despite these limitations, computational approaches can help to identify the targets of enhancer-risk variants. The method developed by Thurman and colleagues was applied to all GWAS loci and predicted gene targets of 419 disease-associated risk variants [20], most of which were located more than 100 kb from the risk SNP. PreSTIGE was utilized to predict gene targets of 122 noncoding loci associated with six immune disorders: rheumatoid arthritis, Crohn’s disease, celiac disease, multiple sclerosis, lupus and ulcerative colitis. Furthermore, at several of the autoimmune-disease-associated loci, the effect of the risk allele on target gene expression was quantified.

## Utilizing expression quantitative trait loci studies to evaluate the impact of enhancer variants

Expression quantitative trait loci (eQTL) studies enable the identification of genetic variants that influence gene expression. eQTL studies involve stratifying a panel of individuals based on their particular SNP genotypes and then determining whether transcript levels differ between individuals based on the specific SNP genotypes. Genome-wide eQTL studies have identified transcripts that differ in expression on the basis of the genotype of the risk allele at GWAS loci. Alternatively, eQTL-based analyses can be applied to candidate interactions between SNPs and gene targets identified through the experimental or computational approaches described above. In both instances, genetic variation inherent in the human population is utilized to reveal the quantitative and directional effect of SNPs on gene expression (that is, the degree to which expression is upregulated or downregulated).

eQTL studies can locate SNPs within a given GWAS locus that influence target transcript levels, but caution must be taken when interpreting results. First, eQTLs, like enhancers, are cell-type specific. Thus, the effect of a SNP on transcription may only occur in disease-relevant cell types [71,72]. Second, the SNP associated with transcript levels may not be the causal SNP: SNPs in LD with the eQTL SNP may be driving the association. Third, the results are correlative and may reflect indirect associations between SNPs and genes. Fourth, the effects on gene expression must be robust in order to be identified over the confounding effects of the genetic background. This poses a challenge for detecting functional variants that have modest effects, as has been proposed for most enhancer variants [7,33,73,74]. Fifth, eQTL analyses rarely consider the combinatorial effects of multiple SNPs at a given locus. Last, because eQTL studies are typically performed on healthy individuals, the impact of the SNP on the quantitative trait may differ in response to disease-specific stimuli. This was observed in a survey of enhancer SNPs associated with prostate cancer. Here, the effect of a SNP on enhancer function was only observed in the presence of the androgen dihydrotestosterone [6]. Additionally, a study by Harismendy and co-workers demonstrated that the chromatin interaction between an enhancer locus associated with coronary artery disease and the gene target *IFNA21* was significantly remodeled by treatment with interferon-γ [31].

## Transcriptional effects of enhancer variants

Studies that delineate the impact of disease-associated enhancer variants (Table 2) reveal the relatively modest effect of enhancer variants on gene expression. The effect of enhancer variants has also been evaluated with massively parallel reporter assays in which the impact of mutations in enhancer sequences is determined through heterologous barcoding and high-throughput sequencing (reviewed in [75]). These high-throughput assays show that most variants that impact transcription induce 1.3- to 2-fold differences in target gene expression [73,74]. These findings align with the notion that enhancers modulate or fine-tune gene expression, analogous to a rheostat. Despite their modest transcriptional effects, enhancer variants can have large effects on downstream phenotypes. As an example, we highlight a SNP (rs12821256) associated with blond hair color in Europeans. This SNP lies in an enhancer that drives *KITLG* expression in developing hair follicles [33]. The blond-hair-associated SNP was shown to reduce enhancer activity by only 22% *in vitro*. Nonetheless, when the blond hair and ancestral alleles were evaluated in transgenic mice, the reduction in enhancer activity associated with the blond hair allele was sufficient to yield mice of visibly lighter coat color than mice generated with the ancestral allele [33]. Whether or not the blond-hair-associated SNP represents a special instance of a more general mechanism in which enhancer variants with modest functional effects have robust phenotypic effects remains to be seen.

**Table 2:** **Functional enhancer studies of GWAS risk loci**

**Disease/trait**	**Reference**	**Lead SNP**	**Proposed functional SNP**	**Gene target**	**How gene target was selected**	**Cell type**	**Data supporting SNP enhancer function**
Blond hair color	Guenther *et al.* [33]	rs12821256	rs12821256	*KITLG*	Phenotype in mouse model	Developing hair follicles, HaCaT karatinocyte cell line	Allele-specific luciferase activity, allele-specific ChIP, effect of SNP on mouse phenotype
Breast cancer	Cowper-Sal lari *et al.* [38]	rs4784227	rs4784227	*TOX3*	3C	MCF7	Binding motif disruption, allele-specific ChIP, allele-specific 3C, allele-specific expression, eQTL
Colorectal cancer	Pomerantz *et al.* [34]	rs6983267	rs6983267	*c-MYC*	3C	Colo205 and LS174T	Allele-specific luciferase activity, allele-specific ChIP
Colorectal cancer	Wright *et al.* [39]	rs6983267	rs6983267	*c-MYC*	3C	DLD1 and HCT116	Allele-specific ChIP, allele-specific expression
Colorectal cancer	Tuupanen *et al.* [36]	rs67491583	rs67491583	*c-MYC*	Nearest gene	HeLa	Binding motif disruption, allele-specific ChIP, allele-specific luciferase activity
Prostate cancer	Wasserman *et al.* [35]	rs6983267	rs6983267	*c-MYC*	Nearest gene	Prostate tissue (mouse)	Allele-specific activity, LacZ enhancer assay (mouse)
Coronary artery disease	Harismendy *et al.* [31]	rs10757278	rs10811656/ rs10757278	*CDKN2B*, *CDKN2BAS*, *IFNA21*, *MTAP*	3C and FISH (*IFNA21*)	HUVEC	Binding motif disruption, allele-specific ChIP
Coronary heart disease	Miller *et al.* [29]	rs12190287	rs12190287	*TCF21*	Nearest gene, eQTL gene	HCASMC	Binding motif disruption, allele-specific luciferase activity, EMSA, allele-specific ChIP and allele-specific expression
Fetal hemoglobin level	Bauer *et al.* [28]	rs1427407, rs7606173	rs1427407, rs7606173	*BCL11A*	3C	Primary human erythroblasts	Binding motif disruption, allele-specific ChIP, allele-specific expression, LacZ enhancer assay (mouse), deletion by TALEN
Multiple sclerosis	Alcina *et al.* [27]	rs658115	rs10877013	*FAM119B*, *AVIL*, *TSFM*, *TSPAN31*	eQTL	LCLs and monocytes	Allele-specific luciferase activity, eQTL
Obesity	Smemo *et al.* [30]	rs9930506	NA	*IRX3*	4C-seq, 3C, ChIA-PET, Hi-C	Whole mouse embryo and adult mouse brain	eQTL mapping
Prostate cancer	Hazelett *et al.* [6]	rs5945619	rs4907792	*NUDT1*	Nearest gene, eQTL gene	LNCaP	Allele-specific luciferase activity, eQTL
Prostate cancer	Hazelett *et al.* [6]	rs10486567	rs10486567	*JAZF1*	Nearest gene	LNCaP	Allele-specific luciferase activity, binding motif disruption
QT interval	Kapoor *et al.* [32]	rs12143842	rs7539120	*NOS1AP*	eQTL gene, genetic association	Cardiac tissues	Allele-specific luciferase activity, eQTL, enhancer assay (zebrafish embryos)
Restless leg syndrome	Spieler *et al.* [37]	rs12469063	rs13469063	*MEIS1*	PreSTIGE prediction method, ChIA-PET, Hi-C	Telencephalon	Allele-specific expression of reporter gene in zebrafish, Allele-specific LacZ (mouse), EMSA, binding motif disruption, effect of decreased gene expression on phenotype
Systemic lupus erythematosus	Wang *et al.* [40]	rs2230926	rs148314165, rs200820567	*TNFAIP3*	3C	LCLs	EMSA, allele-specific luciferase activity, allele-specific 3C
Type 2 diabetes	Gaulton *et al.* [76]	rs7903146	rs7903146	*TCF7L2*	Nearest gene	Pancreatic islet cells	Allele-specific luciferase activity, allele-specific FAIRE

3C, chromosome conformation capture; 4C-seq, circular chromosome conformation capture followed by sequencing; ChIA-PET, chromatin interaction analysis by paired-end tag sequencing; ChIP, chromatin immunoprecipitation; EMSA, electrophoretic mobility shift assay; eQTL, expression quantitative trait loci; FAIRE, formaldehyde-assisted isolation of regulatory elements; FISH, fluorescence *in situ* hybridization; LCLs, lymphoblastoid cell lines; NA, not applicable; SNP, single nucleotide polymorphism.

## Implications for disease and medicine

**Figure 3:**
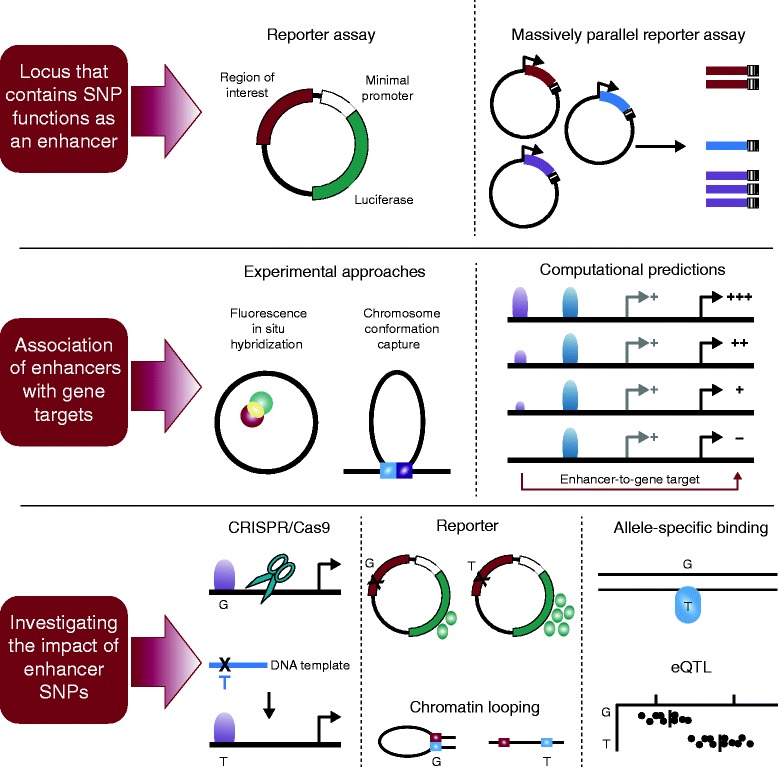
**Interpreting enhancer variants.** Various strategies for interpreting enhancer variants. (Top) Single- or high-throughput reporter assays can be used to test whether a putative enhancer is functional. (Middle) Gene targets of enhancers can be identified through experimental approaches such as fluorescence *in situ* hybridization and chromosome conformation capture assays, or through computational methods. (Bottom) The impact of a single nucleotide polymorphism (SNP) on enhancer function can be evaluated through CRISPR/Cas9-based DNA editing approaches, followed by measures of enhancer activity or target gene expression. The effect of a risk SNP on transcriptional activity and chromatin architecture can be evaluated through reporter assays and chromosome-conformation-capture-based experiments. Effects of the risk SNP on allele-specific expression and transcription factor binding can also be studied through quantitative ChIP and expression studies. Expression quantitative trait loci (eQTL) analysis can be performed to determine the effect of risk SNPs on gene expression levels.

The strategies discussed above (summarized in Figure 3) have been utilized to interpret the transcriptional effects of enhancer variants associated with several traits and common diseases. The continued application of these and other emerging strategies will have important implications for disease and medicine. These studies should not only help to reveal the gene targets of noncoding risk variants, but also provide information on whether these risk variants increase or decrease expression of the target gene. This information will be essential for identifying appropriate therapeutic targets and determining whether inhibitors or activators of these targets would be most effective. Knowledge of gene targets may also reveal pathways that are commonly altered among affected individuals, which could also guide treatment strategies and rational drug design.

## Conclusions and future challenges

**Figure 4:**
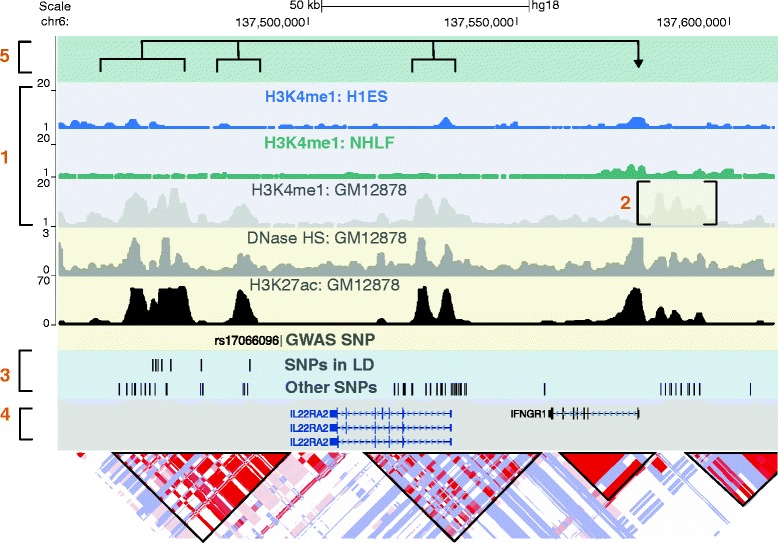
**Future challenges for the functional evaluation of enhancer variants.** The challenges described in the conclusion section are depicted in this hypothetical enhancer locus. Chromatin immunoprecipitation combined with massively parallel DNA sequencing (ChIP-seq) tracks from ENCODE [77] and linkage disequilibrium (LD) plots from HapMap [78,79] are displayed via the UCSC genome browser. Number 1 highlights the challenge of utilizing the proper cell type to assess enhancer activity. Enhancers at this locus are only active in one of the three cell lines depicted. Challenge number 2 is the discrepancy between predicted and validated enhancer function. Shown is a putative enhancer defined by chromatin state that requires experimental validation of its enhancer activity. Challenge number 3 illustrates the large number of single nucleotide polymorphisms (SNPs) in LD that lie in putative enhancer elements, any of which could be functional. Number 4 is the challenge of determining the gene impacted by the enhancer variant. Here, the target of the enhancers at this locus could be *IL22RA2*, *IFNGR1*, or a gene distal to this locus. Number 5 is the complexity of enhancer gene regulation. Here, multiple enhancers each with several associated variants are distributed across the locus. One or a combination of several of the enhancer variants could influence target gene expression. chr, chromosome; GWAS, genome-wide association study; kb, kilobases.

We have reviewed approaches for the identification and interpretation of common-disease-associated variants that impact enhancer function, citing examples in which these methods have been successfully implemented (Figure 3, Table 2). We highlight three main conclusions. First, cell-type-specific enhancer variants are highly prevalent among loci associated with the majority of common diseases identified through GWASs. Second, GWAS-identified enhancer variants are disproportionally enriched in enhancer clusters, which control genes with highly specialized cell-type-specific functions. Third, these enhancer variants can have modest but significant effects on target gene expression, which can have robust effects on phenotype. Thus, interpreting the functional effects of enhancer variants requires rational experiment design that takes these characteristics into account. Furthermore, although current methods have enabled the thorough characterization of enhancer variants at some GWAS loci, high-throughput methods are needed, given the huge number of disease-associated enhancer variants. Here, we discuss additional lessons learned from these studies, and note five remaining challenges (Figure 4).

First, chromatin landscapes vary considerably between cell types and are highly dynamic, capable of altering in response to internal and external environmental stimuli. Given the spacial, temporal, environmental and epigenetic complexity of gene regulation, it is essential that the appropriate human cell type or model is utilized when trying to draw inferences between risk alleles and enhancer elements. Integrating risk variants with the chromatin landscapes of cell types or conditions that are insufficient models for a disorder will likely give misleading results. This is highlighted by eQTL studies. Even in comparisons of relatively similar cell types (monocytes and T cells [72] or B cells and monocytes [71]), noncoding variants that impact expression in one cell type often had no effect in the other cell type. Additionally, in a study of *cis*-regulation in colon cancer, the impact of some SNPs on expression was seen amongst colon cancer samples, but not observed in normal colon from the same patients, implying that the impact of the variant is dependent on disease-specific environmental factors [80]. The effect of noncoding variants on expression was also observed to be strongly context dependent in a study of monocytes under diverse types and durations of stimuli. Fairfax and colleagues demonstrated that 43% of identified eQTLs were associated with an effect on expression only after treatment with immune response stimuli lipopolysaccharide or interferon-γ [81].

Second, there remains a gap between the prediction and functional validation of putative enhancer elements. Thus, if a risk SNP is localized to a putative enhancer element defined through chromatin profiling, it is essential that the putative enhancer is functionally validated. *In vitro* and *in vivo* reporter assays can help in this regard. However, these assays are relatively low throughput and usually involve the use of a general promoter such as SV40 rather than the enhancer’s endogenous promoter, which complicates the interpretation of negative results. Additionally, most genes are regulated by more than one enhancer, yet typically only one enhancer is tested in a reporter assay.

Third, at a given GWAS locus, the SNP with the most significant association (that is, the lowest *P* value) with the disease is usually reported as the ‘lead’ SNP. Except in rare instances, such as the SNP rs6983267, which influences the MYC enhancer and confers risk for multiple cancers [34,35], the SNP with the lowest *P* value is not necessarily causal. Any SNP in LD with the lead SNP may be causal, and there may be dozens to thousands of candidates. Fine mapping studies can help narrow the locus and reduce the number of candidates. Additionally, as discussed above, identifying SNPs that co-localize with enhancer-chromatin features or TFBSs in an appropriate human cell type can help prioritize candidate functional variants [30,38]. Indeed, Claussnitzer and colleagues developed a method, phylogenetic module complexity analysis (PMCA), which utilizes conserved co-occurring TFBS patterns to identify functional regulatory variants [82]. However, hundreds of candidate SNPs may remain even after prioritization, especially when the locus harbors an enhancer cluster. This was illustrated in a recent survey of breast cancer risk loci, which showed that 921 SNPs co-localize with putative enhancers in human mammary epithelial cells across 71 risk loci [8]. Similarly, 663 enhancer SNPs were identified for 77 prostate risk loci [6]. Furthermore, while some enhancer variants influence transcription factor binding [6,28,29,34], SNPs do not necessarily have to reside within a TFBS to influence transcription factor binding or enhancer activity [33,73,74,83]. It is clear that massively parallel reporter assays (discussed above) will be necessary to help distinguish functional variants from those that are passengers.

Fourth, in order to determine the phenotypic effect of an enhancer variant, it is essential that an enhancer variant is demonstrated to influence the levels of its target transcript. The target may be a gene, or could alternatively be a noncoding RNA. However, enhancers frequently regulate multiple genes. Even if the levels of a given transcript correlate with the genotype of an enhancer risk variant, it does not necessarily mean that the correlated gene is causal. Functional assays, and ultimately *in vivo* models, are needed to verify that the gene is directly involved in the development of the disease. CRISPR/Cas9 technology would enable such studies by altering single SNPs in the genome of a model organism while maintaining the native genomic context of the variant. Alternatively, single-site integration of the risk or non-risk alleles into a model organism, as utilized for the enhancer variant associated with blond hair color [33], could be employed. Although CRISPR/Cas9 can be utilized to demonstrate the functional impact of a given variant, the complex phenotypes of many diseases are not easily modeled *in vitro* and thus the determination of causality will often not be trivial.

Lastly, genes are frequently regulated by multiple enhancer elements or clusters of enhancer elements. Thus, the independent effect of a single enhancer or variant may be below the sensitivity threshold of standard assays. This, in addition to the demonstration that multiple enhancer SNPs can act in combination to impact gene expression, suggests that epistatic effects between noncoding variants may play a particularly important role for enhancer loci, especially when enhancer variants of the same gene are inherited independently. The impact of the interaction between SNPs on transcription and ultimately on clinical risk for disease remains to be seen.

We have discussed the strategies for, and challenges associated with, the interpretation of noncoding putative enhancer SNPs as applied to the study of common variants identified by GWAS studies of common diseases and traits. As whole-genome sequencing becomes more prevalent, these same strategies will be necessary to elucidate the impact of rare noncoding mutations and to distinguish damaging from innocuous enhancer alterations.

## Abbreviations

3C: Chromosome conformation capture
4C: Circular chromosome conformation capture
5C: Carbon-copy chromosome conformation capture
CAGE: Cap analysis of gene expression
ChIA-PET: Chromatin interaction analysis by paired-end tag sequencing
ChIP-seq: Chromatin immunoprecipitation with massively parallel DNA sequencing
DHS: DNase I hypersensitivity site
eQTL: Expression quantitative trait loci
FDR: False discovery rate
FISH: Fluorescence *in situ* hybridization
GWAS: Genome-wide association study
H3K27ac: Acetylation of lysine 27 on histone 3 [as an example]
H3K4me: Methylation of lysine 4 on histone 3 [as an example]
IM-PET: Integrated methods for predicting enhancer targets
kb: Kilobases
LD: Linkage disequilibrium
MEV: Multiple enhancer variant
PMCA: Phylogenetic module complexity analysis
PreSTIGE: Predicting specific tissue interactions of genes and enhancers
RNA-seq: RNA sequencing
SNP: Single nucleotide polymorphism
TFBS: Transcription-factor-binding site
VSE: Variant set enrichment
